# IFNγ-induced PD-L1 expression is JAK2 but not JAK1 dependent and its inhibition enhances NK-cetuximab mediated ADCC of HNSCC cells

**DOI:** 10.1186/2051-1426-2-S3-P199

**Published:** 2014-11-06

**Authors:** Fernando Concha-Benavente, Robert L Ferris

**Affiliations:** 1 Department of immunology, Univeristy of Pittsburgh, Pittsburgh, PA, USA; 2 Department of Otolaryngology, University of Pittsburgh Cancer Institute, Pittsburgh, PA, USA; 3 Cancer Immunology Program, University of Pittsburgh Cancer Institute, Pittsburgh, PA, USA

## 

Programmed death ligand 1 (PD-L1) is an immunosuppressive molecule expressed by many cancer types, including a large proportion of head and neck cancers (HNC), and ligation of its receptor, programmed death 1 (PD-1), induces exhaustion of effector T cells. It has been shown that interferon gamma (IFNγ) induces PD-L1 expression in many cancer types including glioblastoma, melanoma, lung and kidney cancer. Importantly, the stimuli and mechanism for PD-L1 upregulation in HNC cells are not well characterized. IFNγ signals through Janus Kinase 1/2 (JAK1/2) heterodimer complex and mediates signal transducer and activator of transcription 1 (STAT1) phosphorylation, leading to type I cytokine expression, upregulation of antigen presentation, and tumor cell recognition by cytolytic T lymphocytes (CTL). We investigated basal PD-L1 expression and the mechanism by which IFNγ signaling upregulates PD-L1 in HNC cells including dependence on JAK/STAT pathway. We observed that IFNγ signaling increased PD-L1 expression in a JAK2 but not JAK1 dependent fashion. In addition, interferon alpha (IFNα), which signals via JAK1/TYK2 did not upregulate PD-L1 expression while still upregulated HLA class I. Specific JAK2 inhibition downregulated NK cell-derived IFNγ induced PD-L1 expression and enhanced cetuximab mediated ADCC. Our data suggest a crucial role for JAK2/STAT1 in IFNγ mediated PD-L1 upregulation. JAK2 inhibition provides a promising strategy to increase tumor cell lysis through maintaining HLA class I while suppressing tumor cell expressed PD-L1 in combination with anti-EGFR cetuximab therapy.

